# Do cortical plasticity mechanisms differ between males and females?

**DOI:** 10.1002/jnr.23850

**Published:** 2016-11-07

**Authors:** James Dachtler, Kevin Fox

**Affiliations:** ^1^ Department of PsychologyDurham UniversityDurhamUnited Kingdom; ^2^ School of BiosciencesCardiff UniversityCardiffUnited Kingdom

**Keywords:** neuropsychiatric conditions, CaMKK, NOS1, estrogen receptor, hippocampus, cerebral cortex

## Abstract

The difference between male and female behavior and male and female susceptibility to a number of neuropsychiatric conditions is not controversial. From a biological perspective, one might expect to see at least some of these differences underpinned by identifiable physical differences in the brain. This Mini‐Review focuses on evidence that plasticity mechanisms differ between males and females and ask at what scale of organization the differences might exist, at the systems level, the circuits level, or the synaptic level. Emerging evidence suggests that plasticity differences may extend to the scale of synaptic mechanisms. In particular, the CaMKK, NOS1 and estrogen receptor pathways show sexual dimorphisms with implications for plasticity in the hippocampus and cerebral cortex. © 2016 The Authors. Journal of Neuroscience Research Published by Wiley Periodicals, Inc.

The difference between male and female animals is unmistakable on the outside, and from a biological perspective one might expect to find many differences on the inside too. It is not controversial that male and female behavior is different both in humans and in less sentient animals and highly likely that many of those behavioral differences can be attributed to differences in brain structure. Over what spatial scale might such differences occur? At the systems level, male and female brains differ in size (Goldstein et al., 2001; Gur et al., 2012) and connectivity (Ingalhalikar et al., 2014); the hypothalamic structures and circuits are different because of their roles in reproduction and hormone regulation (Simerly, 2002; Scott et al., 2015), and sex hormones are known to have effects on the function and development of neuronal circuits (Dohler et al., 1986; Scharfman and MacLusky, 2006). However, is it also possible that differences between the sexes exist on a finer scale, perhaps down to the level of individual synapses and the molecular mechanisms that are involved in synaptic plasticity.

To date, sex differences remain relatively underexplored in neuroscience. The relative lack of exploration may rest partially on practical grounds, such as the belief that biological results are more variable in females than in males, which leads to experimental designs employing only male animals. In a similar vein, because at least twice as many animals would be required to test for a difference in the role of a particular variable between male and female mice, the financial cost of doing so is twice as high and requires twice the time, and the subsequent cost may discourage the practice. In addition to errors of commission there may also be errors of omission. Pogun, noted as recently as 2001 that

Although males and females are unmistakably different, the recognition of sex as a key variable in science and medicine is considered a revolution in some circles. Sex differences transcend reproductive functions, are evident in the structural and functional organization of the brain, and are reflected in group differences in cognitive abilities and behavior. (Pogun, 2001).

Indeed, given the findings in mouse studies that genetic background can have a large effect on learning, plasticity, and behavior (Nguyen et al., 2000; Ranson et al., 2013), it seems almost inevitable that a far less subtle genetic difference between animals, such as an entire chromosome difference, would have some effect. This Mini‐Review explores some of the evidence for differences in plasticity at the cellular level and how those differences might impact learning and memory at the behavioral level. Particular attention is given to the role of estrogen in structural plasticity of dendritic spines and the differing degrees to which nitric oxide synthase (NOS) plays a role in synaptic potentiation in males vs. females. Given recent findings showing that synaptic proteins are major factors in mental health conditions (Hall et al., 2015; Reichelt et al., 2012), we further explore the evidence for sex differences in schizophrenia and autism‐spectrum disorders (ASD) as a possible cause. We begin by summarizing some of the molecular differences reported to date between synapses in males and females.

## MOLECULAR ORGANIZATION OF THE SYNAPSE IN MALES AND FEMALES

**Figure 1:**
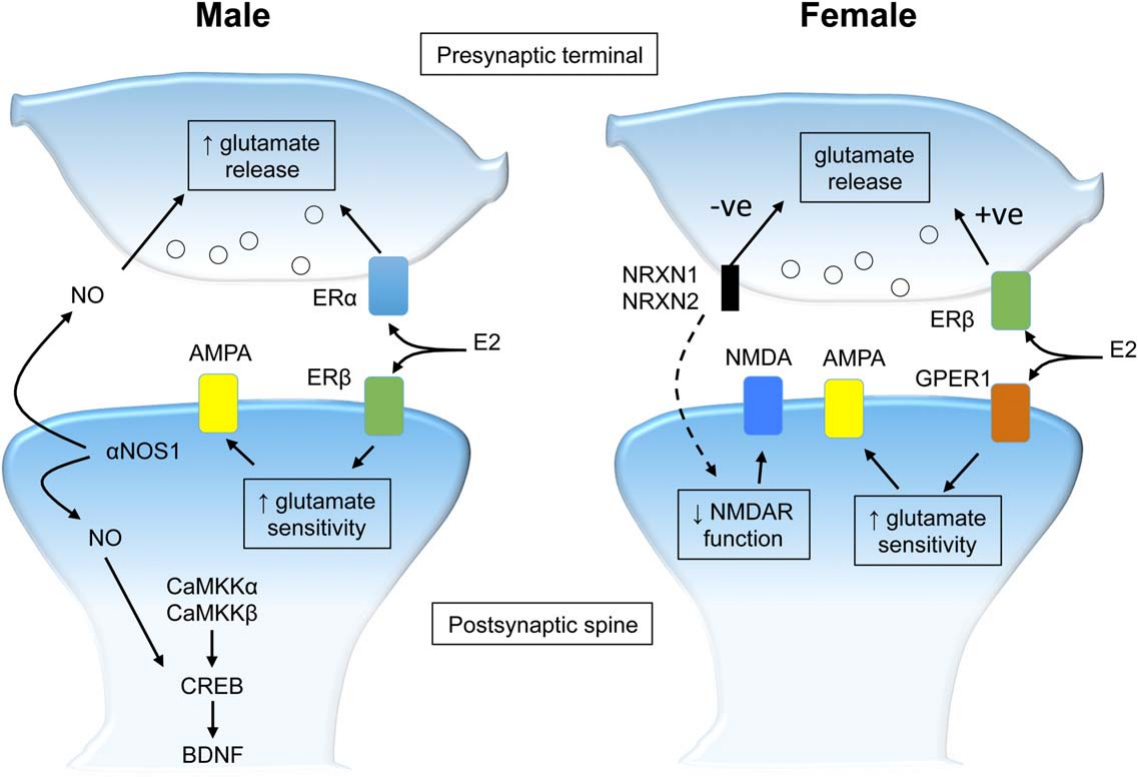
Summary of synaptic plasticity and molecular pathways that differ between the sexes. Neocortical αNOS1 in males has been shown to be involved in in vitro and in vivo synaptic plasticity, with NO acting both pre‐ and postsynaptically. NO and αNOS1 are also more abundant in the male than in the female hippocampus. CaMKKα and CaMKKβ are also more crucial in the male brain for memory tasks, LTP, and CREB transcription. E2 acts via different pre‐ and postsynaptic disposition of oestrogen receptors between the sexes to facilitate increased presynaptic glutamate release and greater postsynaptic glutamate sensitivity; males require presynaptic ERα and postsynaptic ERβ, whereas females employ presynaptic ERβ and postsynaptic GPER1. Deletions of *αNrxn1* and *αNrxn2* have been shown to impair female behaviors, including anxiety, sociability, and memory, and a loss of presynaptic NRXN2 impairs glutamate release and postsynaptic NMDA receptor function in the neocortex. Dashed line represents the effect of mutations in the NRXN genes that would impair synaptic function. NB: the molecules depicted are expressed in males and females but have specific actions or pre‐/postsynaptic locations only in one or the other as shown.

Calcium/calmodulin kinase kinase (CaMKK) signaling has been found to differ in male and female mice, in both behavioral and plasticity studies (Fig. 1). The two isoforms of CaMKK, CaMKKα and CaMKKβ, act by phosphorylating CaMKI and CaMKIV, which in turn modulate the activity of the transcription factor, cAMP‐responsive element binding protein (CREB; Bito et al., 1997), most likely by calcium entry into the cytoplasm through NMDA receptors and L‐type calcium channel (Deisseroth et al., 1998). Mice lacking either CaMKKα or CaMKKβ reveal striking sex differences in tests of behavior and hippocampal synaptic plasticity (for review see Mizuno and Giese, 2010). Male mice lacking CaMKKα have deficits in contextual fear conditioning, which may relate to the lack of upregulation of brain‐derived neurotrophic factor (BDNF) by CaMKKα in males that would normally accompany this task in females (Mizuno et al., 2006). Male CaMKKβ‐deficient mice are impaired in spatial learning and lack hippocampal CREB activation compared with female null mice and also lack late‐phase hippocampal long‐term potentiation (LTP; Mizuno et al., 2007). Baseline sex differences in CREB signaling also exist, with male neonatal rodents having greater phosphorylated CREB expression in hippocampal CA1 (Auger et al., 2001). It is known that CREB is involved in neocortical experience‐dependent plasticity (Glazewski et al., 1999; Barth et al., 2000), but it is not known whether the hippocampal differences in CaMKK and CREB signaling extend to neocortical circuits. These studies provide clear evidence for the basis of sex differences in synaptic plasticity, but further work is required to 1) determine whether CaMKK differences generalize to other brain regions, including the cortex, and 2) identify further sex‐related protein candidates for differences in plasticity.

## SEX DIFFERENTIATION OF THE ROLE OF α‐NITRIC OXIDE SYNTHASE‐1 IN CORTICAL PLASTICITY

Studies in the barrel cortex have shown that both pre‐ and postsynaptic mechanism are activated during expression of cortical synaptic potentiation (Hardingham and Fox, 2006). The postsynaptic aspect of potentiation is dependent on αCaMKII autophosphorylation and GluA1 (GluR1), whereas the presynaptic component depends on postsynaptically located αNOS1 (Hardingham et al., 2003; Hardingham and Fox, 2006). Studies from hippocampus, cerebral cortex, and several other brain structures all suggest that nitric oxide is involved in increasing transmitter release, most likely through a coordinated and synergistic action on several components of the presynaptic release machinery (Hardingham et al., 2013). Theoretically, both activity‐dependent isoforms of nitric oxide synthase (NOS1 and NOS3) could be the source of the nitric oxide signal, and indeed there is evidence that NOS3 and αNOS1 are involved in providing a tonic and phasic release of nitric oxide, respectively (Hopper and Garthwaite, 2006).

**Figure 2:**
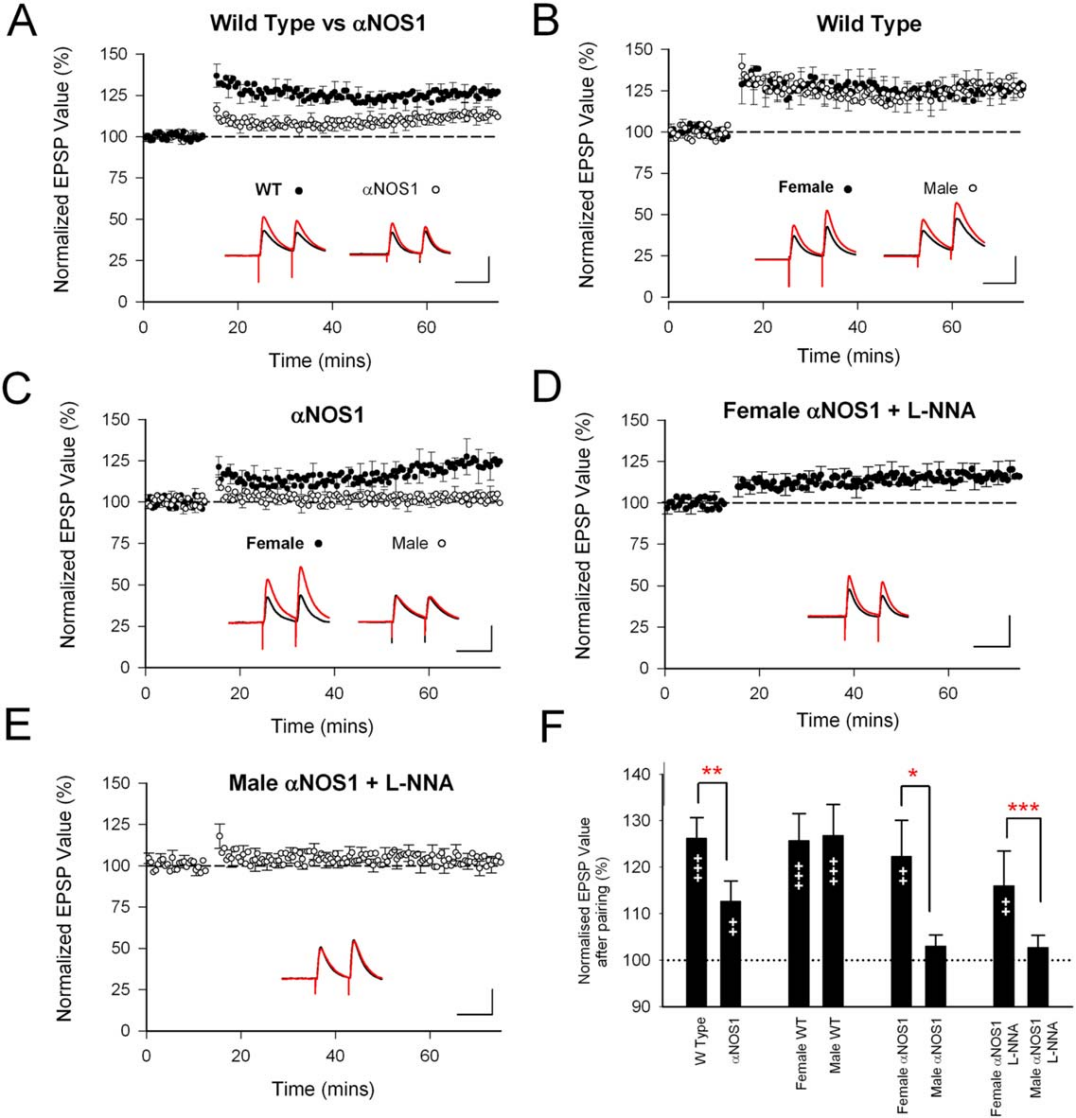
LTP is reduced in αNOS1 knockouts in a sex‐specific manner. **A**: LTP is reduced in αNOS1 knockouts compared with wild types (WTs; sexes combined). **B**: Male and female WTs showed similar magnitudes of LTP. **C**: Male αNOS1s show no significant LTP, whereas female αNOS1 knockouts do show LTP. L‐NNA has no effect on LTP in female αNOS1 knockouts (**D**) or male αNOS1 knockouts (**E**), which already lack LTP. **F**: Average level of potentiation observed at 60 min, showing within‐group significance (††*P* < 0.01, †††*P* < 0.001, paired t‐test) and comparisons between genotypes or sexes (*P < 0.05, **P < 0.01, ***P < 0.005). Scale bar = 100 msec/5 mV for example paired pulse EPSPs. Reproduced from the Journal of Neuroscience Dachtler et al. (2012) with permission.

The phasic component of nitric oxide release is calmodulin‐ and αNOS1‐dependent and can be triggered by NMDA receptor activation. Early indications that the function of αNOS1 might be different between males and females came from studies showing that knocking out αNOS1 confers some neuroprotection from ischemic damage produced by stroke in the male brain but has no protective effect on the female brain (Huang et al., 1994; McCullough et al., 2005). Subsequently, it was found that αNOS1 is also necessary for LTP in male but not female mice (Dachtler et al., 2012). The residual LTP in female mice is not susceptible to a general NOS antagonist, suggesting that the main component of LTP does not rely on nitric oxide signaling at all in female animals (Fig. 2). The dependence of male plasticity on NO may relate to baseline differences between the sexes. Females have, within the hippocampus, less abundant NO and reduced NOS1 expression compared with males, although the application of estradiol increases hippocampal NOS1 expression (Hu et al., 2012). Therefore, a possible explanation for this sex difference is that females lack available NO for the induction of plasticity and thus rely on other molecular pathways instead. It is conceivable that the sex differences in plasticity and susceptibility to stroke damage are related. If nitric oxide is released during ischemic damage, it would tend to potentiate excitatory transmission in the male brain, leading to greater NMDA receptor activation, greater calcium entry, and greater excitotoxic damage.

Experience‐dependent potentiation in the somatosensory cortex depends on many of the same factors as LTP. For example, both αNOS1 and GluA1 are required for pre‐ and postsynaptic components of LTP, respectively, and both are important for experience‐dependent plasticity in the barrel cortex (Dachtler et al., 2011). Knocking out both GluA1 and αNOS1 abolishes experience‐dependent potentiation in the cortex, whereas knocking out either gene individually produces a reduction in but not a complete elimination of plasticity (Dachtler et al. 2011). Subsequent, experiments showed that the αNOS1 component of experience‐dependent plasticity was also sex dependent, as with LTP (Dachtler et al., 2012). Depriving mice of all but one whisker for a period of 3–4 weeks normally results in expansion of the spared whisker domain within the barrel cortex, such that neurons located in barrels surrounding the spared whisker's home barrel are far more sensitive to spared whisker stimulation than in a normal animal (Glazewski et al., 1996). This process is dependent on intracortical circuits and, crucially, on excitatory connections between cortical columns (Fox, 1994; Glazewski et al., 2000). Studies comparing experience‐dependent plasticity in male and female αNOS1 knockout mice showed that experience‐dependent potentiation was almost completely absent in αNOS1^–/–^ males and intact in αNOS1^–/–^ females (Dachtler et al., 2012). Taken together with the lack of LTP in the male αNOS1 knockouts, these findings suggest either that potentiation is αNOS1 dependent only in male mice or that females have an alternate (or compensating) mechanism that comes into play when αNOS1 is knocked out and this compensation system is absent in males. In either case, these studies provide evidence for the theory that cortical synaptic plasticity is differentiated at the level of the synapse between the two sexes.

## ROLE OF ESTROGEN IN SYNAPTIC PLASTICITY

CaMKK and αNOS1 are examples of distinct, sexually dimorphic molecular pathways underpinning synaptic plasticity. However, the most obvious difference between the sexes is the presence of circulating estrogen (predominantly as 17β‐estradiol [E2]), a molecule known to affect synaptic plasticity directly (Cordoba Montoya and Carrer, 1997). High levels of estrogen are present in the female brain from circulating hormone, and noncirculating estrogen is also present in the male brain, albeit at far lower concentrations, where testosterone acts as a precursor to estrogen by aromatase catalysis (Gillies and McArthur, 2010).

### Does Estrogen Have a Direct Role in Synaptic Plasticity?

Warren et al. (1995) performed a simple assay to examine whether estrogen has a role in LTP by testing females at different points in their estrous cycle. They found that, when females were at proestrus, the magnitude of hippocampal LTP was greater than that at either diestrus or estrus (Warren et al., 1995). Prior to this study, others had noted that the abundance of E2 was correlated with dendritic spine plasticity in the hippocampus; spine density was 32% lower at estrus compared with the proestrus phase of the cycle (Woolley and McEwen, 1992). The E2‐dependent increase in spine density at proestrus could be stabilized by NMDA receptors; it was prevented by NMDA receptor antagonists, whereas AMPA or muscarinic receptor antagonists had no effect (Woolley and McEwen, 1994). E2 acted to increase the sensitivity of synapses to NMDA receptor‐mediated input (Woolley et al., 1997); LTP increased only when both spine density increased and there was an enhancement of NMDA receptor transmission relative to AMPA receptor transmission (Smith and McMahon, 2005). Indeed, although E2 was found to cause spinogenesis, synapses remained “silent” unless NMDA receptor activation occurred, allowing the spine to stabilize (Srivastava et al., 2008). Specifically, E2‐dependent facilitation of LTP appears to act through GluN2B‐containing (also known as *NR2B*) NMDA receptors (Smith and McMahon, 2006). Overall, acute treatment of E2 causes increases in spine density and facilitation of LTP through GluN2B receptors, which could be linked to the estrus cycle in females.

### Sex Difference In Estrogen and Plasticity

E2 treatment in slices of hippocampal CA1 acutely potentiates glutamatergic synapses of both sexes. Although males and females arrive at the same plasticity outcome of E2 treatment, the mechanisms by which they do it vary. The compound WAY20070 is an agonist of the β form of the estrogen receptor (ERβ) and causes an increase in the frequency of miniature excitatory postsynaptic currents (mEPSCs) in females but not males and, conversely, increases the amplitude of mEPSCs in males but not females. An ERα agonist (PPT) increases mEPSC frequency only in males (Oberlander, 2016). Hence, E2 acts via distinct estrogen receptors at pre‐ and postsynaptic locations. Furthermore, an agonist to the G‐protein‐coupled estrogen receptor‐1 (GPER1) causes increases in mEPSC amplitude in females but not in males (Oberlander, 2016). Therefore, at glutamateric synapses, in females, E2 facilitates potentiation by postsynaptic GPER1 and presynaptic ERβ, whereas, in males, E2 acts through postsynaptic ERβ and presynaptic ERα (Fig. 1). Currently it is unclear what advantage this sex difference would convey, and whether the recruitment of other downstream elements of the pre‐ and postsynaptic machinery (such as NOS) are consequently differentially modulated.

Recent studies have also demonstrated a role for E2 in inhibition and hve begun to reveal not only that is this sex specific but that E2 acts through distinct molecular pathways. Acute E2 application to hippocampal slices has revealed a rapid suppression of GABAergic inhibitory synaptic transmission at perisomatic inputs to pyramidal cells within the hippocampal CA1, specifically through a molecular cascade including the α form of the estrogen receptor (ERα), metabotrophic glutamate receptor 1 (mGluR1), and endocannabinoid receptor 1 (Huang and Woolley 2012). Remarkably, this inhibitory effect was evident only in females; E2 had no effect on inhibitory postsynaptic currents (IPSCs) in males (Huang and Woolley 2012). Further work revealed this was because E2 promotes an ERα–mGluR1 interaction only in females, which in turn stimulates production of phospholipase C and inositol triphosphate (IP_3_), leading to postsynaptic endocannabinoid release (Tabatadze, 2015).

## Is Circulating Estrogen the Source of the Sex Difference?

Theoretically, the sex differences in ERs discussed above could account for different effects of estrogen from the common source of aromatase, present in males and females. However, several studies suggest that circulating estrogen is in practice the cause of the spinogenic differences in hippocampal plasticity. First, E2 levels are very much lower in males (below detection limits [0.07pM]) than in females. Second, in vivo, E2 increases spine density only in females. Third, spinogenesis still occurs if aromatase is blocked with tetrazole in cycling females, but this has no effect in males (Fester et al., 2012). Finally, E2 increases LTP sensitivity only in females (Vierk et al., 2015). Although these findings do not preclude aromatase‐generated E2 as a determinant of other sex differences, they do imply that the major difference in LTP between the sexes can be attributed to the action of circulating hormone.

## SEX DIFFERENCES IN BEHAVIOR AND NEUROPSYCHIATRIC DISORDERS INVOLVING THE CORTEX

Behavior is the final output of all the upstream synaptic functions, so sex differences in behavior can reveal insights into how cortical activity differs between males and females. Likewise, aberrations in behavior caused by certain psychiatric conditions tend to be sex specific in their expression, which can provide insight into which cortical circuits and synaptic mechanisms differ between the sexes.

### Sex Differences in Cortex‐Dependent Behaviors

Males and females show differences in cortical connectivity that develop early in adolescence (Ingalhalikar et al., 2014). In particular, prefrontal cortical areas appear to be more strongly linked across hemispheres in females and more strongly linked within hemispheres in males (Ingalhalikar et al., 2014). Prefrontal cortical function also differs between males and females. The Iowa gambling task (IGT) probes probabilistic learning; the subject is asked to win as much money as possible without initial knowledge of a winning strategy. A winning strategy is to select cards from a pack that yields smaller rewards but overall monetary gain rather than packs containing larger rewards but overall monetary loss. Studies show that the winning strategy is adopted before the subject is aware of it (Bechara et al., 1997). fMRI measurements show that the IGT engages the dorsolateral prefrontal cortex, the insula and posterior cingulate cortex, and the orbitofrontal and ventromedial prefrontal cortex (Li et al., 2010). Males consistently perform better than females by learning more quickly to avoid the card selections that cause the greater monetary punishment (van den Bos et al., 2013). fMRI studies suggest that male and females engage different parts of the cortex during the IGT, which might explain the sex difference. Males show activity in the left and right lateral orbitofrontal cortex and the right dorsolateral prefrontal cortex, whereas females show activation of the left dorsolateral prefrontal cortex, left frontal gyrus, and temporal lobe (Bolla et al., 2004). In a rat‐based version of the IGT, performance is modulated by both serotonin and dopamine signaling (Zeeb et al., 2009), and, similarly to humans, male rats perform better than females (van den Bos et al., 2012). Further work is required to understand why males and females engage different parts of their brains during certain cortical behaviors and whether each sex employs different neurotransmitters and different synaptic plasticity mechanisms. Nevertheless, these studies do emphasize that, in addition to the sex differences seen at the synaptic scale, sex differences may also manifest themselves at the systems level, and indeed the two may interact. The sex differences seen in prefrontal cortex may be a factor in sex differences seen in psychiatric diseases, as discussed below.

### Sex Differences in Cortical Function in Neurobehavioral Disorders

#### Schizophrenia

Schizophrenia is a psychiatric disorder that has a clear association with cortical impairments. In particular, changes to the structure and function of the prefrontal cortex are particularly characteristic of schizophrenia, with studies noting cortical thinning (Kuperberg et al., 2003), alterations in neural density (Heckers, 1997), and reduced activity during prefrontal‐dependent tasks (Weinberger et al., 1986). The risk ratio of developing schizophrenia in males compared with females is approximately 1.4 (Aleman et al., 2003), with males tending to develop schizophrenia at an earlier age (Faraone et al., 1994), suggesting that there could be underlying sex differences in cortical abnormalities that affect the disease expression. Indeed, there is evidence for this hypothesis. Gross anatomical sex differences in the schizophrenic brain as detected by MRI remain controversial (in part because of the lack of testing sex‐balanced groups), but studies suggest female‐specific reductions and male‐specific enlargements in white matter volume within the occipitoparietal lobe (Highley et al., 2003) and reductions in temporal lobe (Bryant et al., 1999) and anterior cingulate cortex volume (Bryant et al., 1999) in male patients. Differences in molecular signaling may also be sex dependent. Altered expression of GABAergic genes in the anterior cingulate cortex of schizophrenic patients varies by sex; in males, GABA‐Aα5, GABA‐Aβ1, and GABA‐Aε had reduced expression, whereas, in females, GABA‐Aβ1 and GAD67 were upregulated (Bristow et al., 2015).

Mouse models of schizophrenia have revealed sex‐dependent effects. Female mice carrying a missense mutation within the C‐terminal of Disrupted‐in‐Schizophrenia‐1 (*Disc1*) had altered sociability, hyperlocomotion, and heightened anxiety (Dachtler et al., 2016), a profile similar to that of elderly females harboring single‐nucleotide polymorphisms within *DISC1* (Harris et al., 2010). A separate mouse model expressing inducible truncated *Disc1* also showed sex‐specific behaviors, with males having enhanced spontaneous locomotor activity and alterations in social interaction and females having deficient reference spatial memory in the Morris water maze (Pletnikov et al., 2008). Recent evidence has highlighted that, without the normal expression of *DISC1* during development, adult in vivo and in vitro synaptic plasticity is impaired in both the somatosensory (Greenhill et al., 2015) and the visual cortex (Tropea et al., 2016). Hence, *DISC1* may contribute to the genesis of sex differences in schizophrenia.

#### Autism‐spectrum disorders

Autism is a heterogeneous cluster of behavioral abnormalities, which correspondingly has a differing diagnosis depending on the severity of these symptoms. Males consistently have a substantially greater incidence of autism compared with females, with male:female ratios up to 15:1, although on average this is closer to 4:1 (Wing, 1981). A possible cause of this sex difference could lie in hormonal effects during development and has been discussed in terms of the “extreme male brain” theory (Baron‐Cohen et al., 2011), which itself is underpinned by the fetal testosterone theory and the X chromosome theory. Several recent reviews have been published on this topic (Werling and Geschwind, 2013; Schaafsma and Pfaff, 2014; Mottron et al., 2015), so here we focus instead on other possible factors that might contribute to sex differences in autism.

Research into the causative factors explaining the male bias in autism have pointed toward sex differences in the structure and function of the cortex. It is well established that brain enlargement occurs in autism, with enlargement of the cerebral cortex evident before the second year of life (Hazlett et al., 2011). Widespread differences in cortical gray matter have been observed across the frontal (Abell et al., 1999; McAlonan et al., 2002), parietal (McAlonan et al., 2002), and temporal (Boddaert et al., 2004; Hazlett et al., 2011) lobes. However, some of these differences vary by sex. In comparing autistic girls and boys, significant differences in gray matter were observed in the motor cortex, supplementary motor area, insular cortex, and amygdala (Supekar and Menon, 2015). In males but not females, a significant negative correlation has been observed between behavioral autism traits and default‐mode functional connectivity of the medial prefrontal cortex (Jung et al., 2015) along with reduced gyrification in the ventromedial prefrontal and orbitofrontal cortex (Nordahl et al., 2015). Diffusion tenor imaging‐derived fiber tracking has revealed subtle differences in the corpus callosum of preschool‐aged children with autism. Males have a smaller callosal projection region to the orbitofrontal cortex, although females had a smaller region projecting to the anterior frontal cortex (Nordahl et al., 2015).

Sex differences in cortical function in autism may pertain to altered gene expression at the synapse. Retinoic acid‐related orphan receptor alpha (*RORA*), a gene found to be downregulated in autistic patients (Nguyen et al., 2010), is upregulated by E2 but, if the protein is deficient, will cause an accumulation of testosterone through the lack of suppression of *CYP19A1* activity (encoding aromatase; Sarachana et al., 2011). The regulation of *RORA* and its transcriptional targets, including *CYP19A1*, is tightly regulated in the male cortex but less so in females (Hu et al., 2015), suggesting that *RORA* dysregulation could have greater impact on E2 and testosterone regulation and aromatase activity in cortex of male autism patients. Dysregulation of synaptic genes in autism mouse models has revealed sex differences in autistic‐like phenotypes. Mutations within the neurexin genes (*NRXN1–3*) have been widely associated with autism (Chen et al., 2014), and female mice with deletions of *αNrxn1* exhibit impairments in fear learning, although female *αNrxn2* knockout mice have reduced sociability and increased repetitive behaviors (Born et al., 2015; Dachtler et al., 2015). Deficiency of *Nrxn2* has been shown to impair NMDA receptor function, short‐term plasticity, and excitatory transmitter release in cortical layer V cells (Born et al., 2015), implying that some of the behavioral effects of *NRXN* deletion could be related to impaired cortical function.

Taken together, these studies show that differences at the synaptic level play a part in some of the behavioral impairments associated with autism between males and females, in addition to differences in cortical structure and connectivity.

## CONCLUSIONS

We began this Mini‐Review by posing the question of whether sex differences might extend down to the synaptic scale and whether plasticity might differ between males and females as a result. The current literature yields a number of examples of such differences at the synaptic level, most notably the CaMKK pathway, the NOS1 pathway, and the differential effect of circulating estrogen on synaptogenisis, NOS1, and GABAergic transmission. The NOS1, CaMKK, and estrogen effects are all capable of influencing the exact nature of plasticity and hence of affecting learning, memory, and cognition. It remains to be determined the extent to which these are general cortical mechanistic differences, however, because some features such as the effects of estrogen on spinogenesis have thus far been documented only in the hippocampus.

Recent studies have shown that many of the risk factors for psychiatric diseases affect synaptic proteins. Furthermore, studies are now emerging showing that plasticity is altered or impaired in mouse models of psychiatric diseases. For example, a transient disruption of normal DISC1 activity during a critical period of early development affects cortical plasticity into adulthood. It is well known that schizophrenia and ASD show different prevalence across males and females. We therefore raise the possibility here that some of the differential susceptibility to neuropsychiatric disorders between males and females may arise from sex differences in the plasticity mechanisms that are perturbed in those conditions.

## ROLE OF AUTHORS

JD and KF both wrote the review, JD conducted the original research on autism and NOS1 as cited, KF conceived and designed experiments for the NOS1 experiments as cited.
